# Impact of apparent diffusion coefficient on prognosis of early hepatocellular carcinoma: a case control study

**DOI:** 10.1186/s12893-022-01892-6

**Published:** 2023-01-11

**Authors:** Shinichiro Yamada, Yuji Morine, Tetsuya Ikemoto, Yu Saito, Hiroki Teraoku, Yuhei Waki, Chiharu Nakasu, Mitsuo Shimada

**Affiliations:** Department of Digestive and Transplant Surgery, Tokushima University Hospital, 3-18-15 Kuramoto-cho, Tokushima City, Tokushima 770-8503 Japan

**Keywords:** Magnetic resonance imaging, Apparent diffusion coefficient, Hepatocellular carcinoma, Prognostic prediction

## Abstract

**Background:**

We investigated the usefulness of apparent diffusion coefficients (ADC) from diffusion-weighted images (DWI) obtained using magnetic resonance imaging (MRI) for prognosis of early hepatocellular carcinoma (HCC): Barcelona Clinic Liver Cancer (BCLC) stage 0 and A.

**Methods:**

We enrolled 102 patients who had undergone surgical resection for early HCC: BCLC stage 0 and A, and calculated their minimum ADC using DWI-MRI. We divided patients into ADC^High^ (n = 72) and ADC^Low^ (n = 30) groups, and compared clinicopathological factors between the two groups.

**Results:**

The ADC^Low^ group showed higher protein induced by vitamin K absence-II (PIVKA-II) levels (p = 0.02) compared with the ADC^High^ group. In overall survival, the ADC^Low^ group showed significantly worse prognosis than the ADC^High^ group (p < 0.01). Univariate analysis identified multiple tumors, infiltrative growth, high PIVKA-II, and low ADC value as prognostic factors. Multivariate analysis identified infiltrative growth and low ADC value as an independent prognostic factor.

**Conclusion:**

ADC values can be used to estimate the prognosis of early HCC.

## Background

Hepatocellular carcinoma (HCC) is the fourth leading cause of cancer-related deaths worldwide [1]. Recently, the treatment strategy for HCC has been strictly defined in Japan [2] on the basis of tumor status, vessel invasion, extrahepatic metastasis and liver function. In this strategy, for solitary tumors or no more than three tumors, none larger than 3 cm without macroscopic vessel invasion, surgical resection, ablation therapy and transarterial embolization are recommended [2]. On the other hand, there are many criteria for treatment of HCC in the world. Most widely used criteria is classification of Barcelona Clinic Liver Cancer (BCLC) [3]. BCLC classification includes not only tumor status, vessel invasion and extrahepatic metastasis, but also performance status, portal vein pressure and bilirubin level. In very early and early stage (stage 0 and A) of BCLC classification, resection, ablation therapy, ethanol injection or liver transplantation are recommended [4]. In recent years, some studies have reported that surgical resection shows better outcomes compared with other therapies [5, 6]. However, prognosis of early HCC after surgical resection varies. In some patients, tumors recur and progress very quickly [7]. This may be due to tumor heterogeneity, with some tumors manifesting highly aggressive characteristics [8]. Therefore, modern practices would benefit from preoperative methods that evaluate tumor characteristics accurately.

Recent advances in diffusion-weighted imaging (DWI) of magnetic resonance imaging (MRI) have radically improved detection and characterization of solid tumors [9]. DWI is a functional MRI technique that can evaluate water molecule diffusion and assess the histopathological condition of tissues and organs, using a high scan speed without a contrast agent. Apparent diffusion coefficient (ADC) values provide a quantitative image of diffusion characteristics [10]. More specifically, ADC values decrease in areas where diffusion is restricted, such as rich stroma or tissues with high cellularity. Recently, ADC values have been shown to predict prognosis in various cancers [11, 12]. We reported that ADC values can predict prognosis of intrahepatic cholangiocarcinoma [13]. ADC values can also be used to estimate histological grade of HCC [14]. Furthermore, some studies have reported that ADC values can predict early recurrence [15] and metastatic recurrence [16] after curative resection of HCC. However, to our knowledge, no studies have reported prognostic prediction of overall survival using ADC values in early HCC; BCLC stage 0 and A.

This study therefore investigated the usefulness of ADC values in prognostic prediction of early HCC; BCLC stage 0 and A.

## Methods

### Patients and MRI imaging

In this study, we enrolled 112 patients who underwent surgical resection for early HCC of BCLC stage 0 and A at the Department of Surgery, Tokushima University Hospital, between January 2004 and December 2020. We included only patients who (a) had undergone preoperative abdominal MRI, including DWI, within 4 weeks before surgery; (b) had no history of previous treatment prior to surgery, and were without extrahepatic metastasis; and (c) had pathologically proven HCC. For gallbladder cancer, we defined pathological parameters, morphological parameters, and Japanese Tumor-Node-Metastasis stage, in accordance with the Liver Cancer Study Group of Japan [17].

**Fig. 1:**
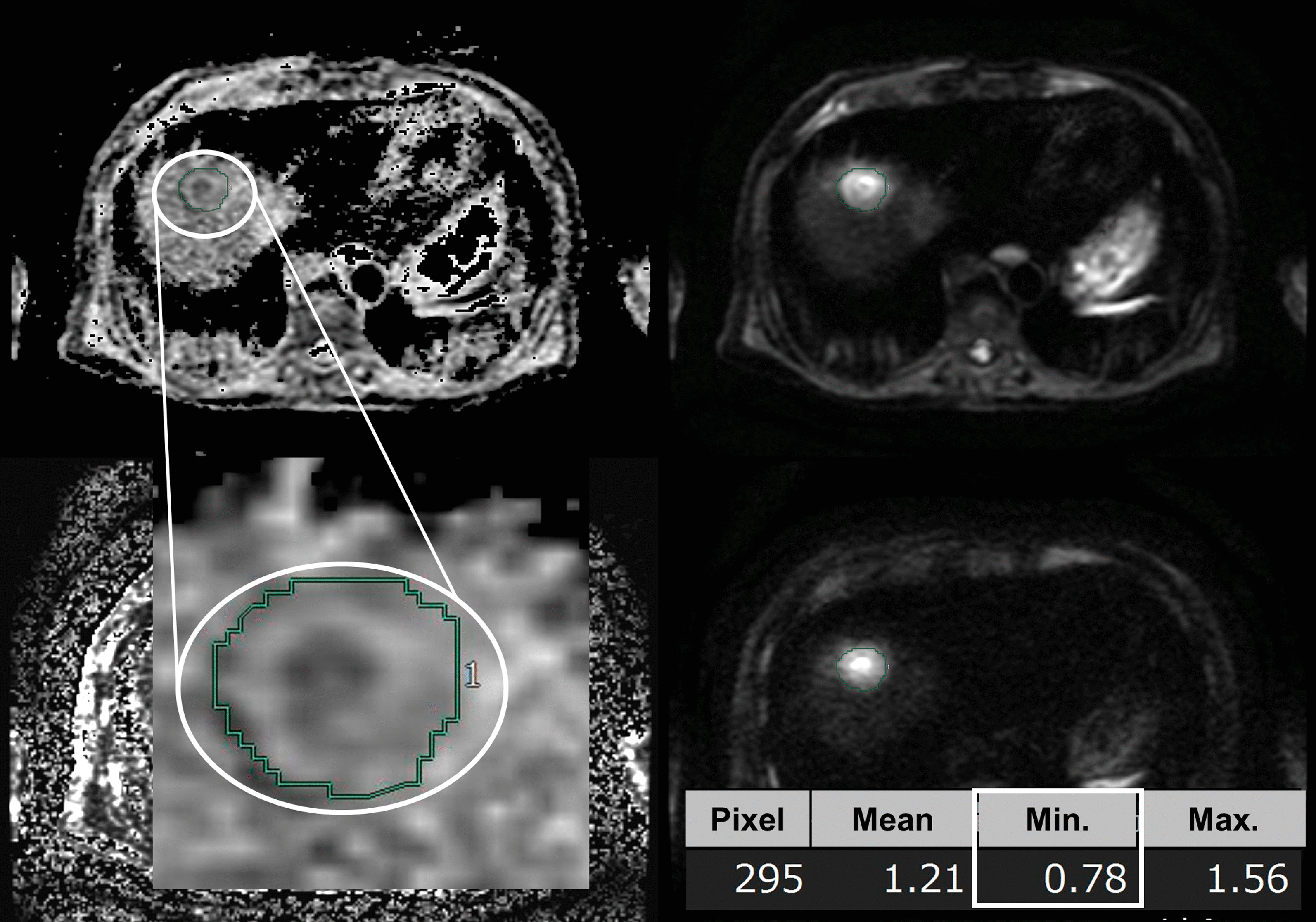
Calculation of ADC values using SYNAPSE VINCENT. This application automatically calculates mean, minimum, and maximum values within free-form green outlines (manual tracing)

**Fig. 2:**
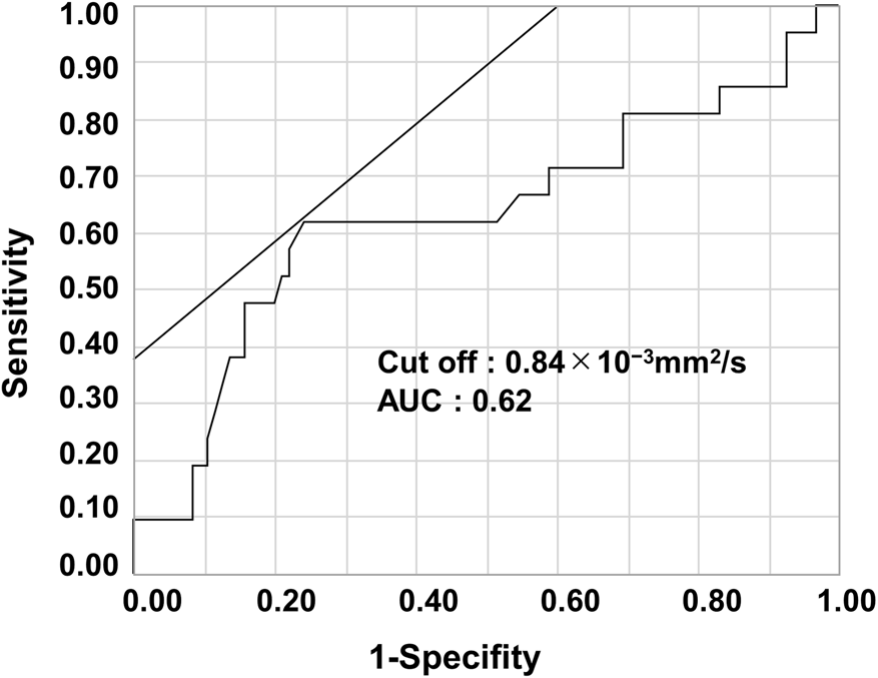
ROC analysis to determine appropriate cut-off value for minimum ADC values. Cut-off value was 0.84 × 10^− 3^mm^2^/s with area under the curve of 0.62

We obtained MR images using 1.5-T superconducting units (Signa HDe/Explorer, GE Medical Systems, Milwaukee, WI, USA) with 8-channel phased-array coil. We recorded fast spin-echo T2-weighted images and DWI (b = 0, 20, 800 s/mm^2^). We measured minimum ADC values (×10^− 3^ mm^2^/s) associated with tumors within regions of interest (ROI) using manual tracing from ADC maps on Synapse Vincent (Fujifilm Medical, Tokyo, Japan) [13]. Synapse Vincent can calculate mean, minimum, and maximum values from free-form green outlines automatically (Fig. 1). In this study, we used minimum ADC values because several studies have reported a significant correlation between such values and histological grade in HCC [14, 18]. Patients were divided into two groups: ADC^High^ group (*n* = 82) and ADC^Low^ group (*n* = 34). The cut-off value of 0.84 × 10^− 3^ mm^2^/s was defined via ROC analysis (Fig. 2). This study was approved by the ethics committee of Tokushima University Hospital.

### Statistical analysis

Continuous variables are presented as min-max (median) and the unpaired Mann–Whitney U test was used. Other factors are presented as number and Fisher’s exact test was used for differentiation. We generated overall survival (OS) and disease-free survival (DFS) curves using the Kaplan–Meier method, and compared differences using the log-rank test. Variables with a p value < 0.1 in univariate analyses were included in the multivariate survival analysis by using a Cox proportional-hazards model [19]. For all statistical analyses, p *<* 0.05 was considered significant. All statistical analyses were performed using statistical software (JMP 8.0.1, SAS Campus Drive, Cary, NC, USA).

## Results

### Clinicopathological features

Table 1 summarizes clinicopathological variables associated with both ADC^Low^ and ADC^High^ groups. Patients in the ADC^Low^ group showed significantly higher protein induced by vitamin K absence-II (PIVKA-II) level (p = 0.02). However, the two groups did not significantly differ with respect to age, sex, liver function, operation procedure, pathological findings of non-cancerous liver tissue and tumor factors such as tumor size, number, portal invasion, and stage.

**Table 1:** Patient characteristics in the low ADC group and in the high ADC group

Variable	ADC low (n = 34)	ADC high (n = 82)	*P*-value
Age (years)	38–84 (70)	38–90 (69)	0.96
Sex (M/F)	25/9	60/22	0.97
Albumin (mg/dl)	3.0-4.8 (3.9)	2.5–4.8 (4.0)	0.12
Platelet (× 10^4^ /µl)	5.5–130 (16.3)	4.6–39.8 (18.4)	0.44
ICG clearance test (%)	2.8–31.3 (10.1)	2.2–53 (9.1)	0.44
Child-Pugh classification (A/B)	34/0	81/1	0.40
Hepatitis (B/ C/non B non C)	8/10/16	18/30/34	0.75
AFP (< 100/≥ 100 ng/ml)	30/4	70/12	0.68
PIVKA-II (< 400/≥ 400 mAU/ml)	22/12	70/12	0.02
Maximum diameter (cm)	1–12 (3)	0.9–10 (3)	0.96
Tumor number (single/multiple)	28/6	71/11	0.56
Growth type (Eg/Ig)	26/8	71/11	0.19
Portal invasion (−/+)	32/2	81/1	0.18
Stage (I/II/III)	4/25/5	14/60/8	0.62
Type of hepatectomy (minor/major)	29/5	67/15	0.64
Pathological finding (NL/CH/LC)	4/17/13	14/52/16	0.11

*ICG* indocyanine green, *AFP* alpha-fetoprotein, *PIVKA-II* protein induced by vitamin-K absence II, *Eg* expansive growth, *Ig* infiltrative growth

*NL* normal liver, *CH* chronic hepatitis, *LC* liver cirrhosis

Continuous variables are presented as min–max (median) and the unpaired Mann–Whitney U test was used. Other factors are presented as number. Fisher’s exact test was used for Differentiation

### Overall and disease-free survival rates

**Fig. 3:**
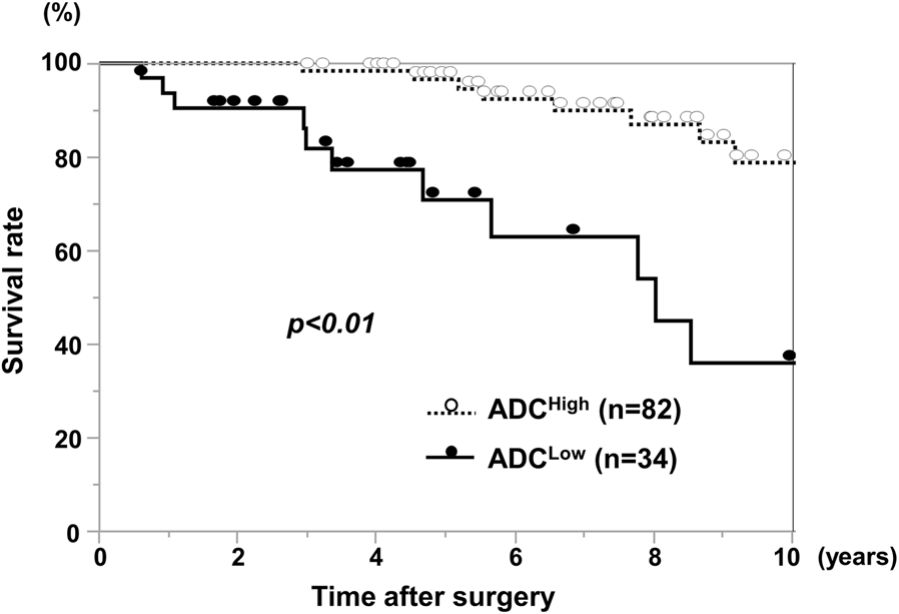
Overall survival rate of ADC^Low^ and ADC^High^ groups. The ADC^Low^ group showed significantly worse prognosis than the ADC^High^ group (p < 0.01)

**Fig. 4:**
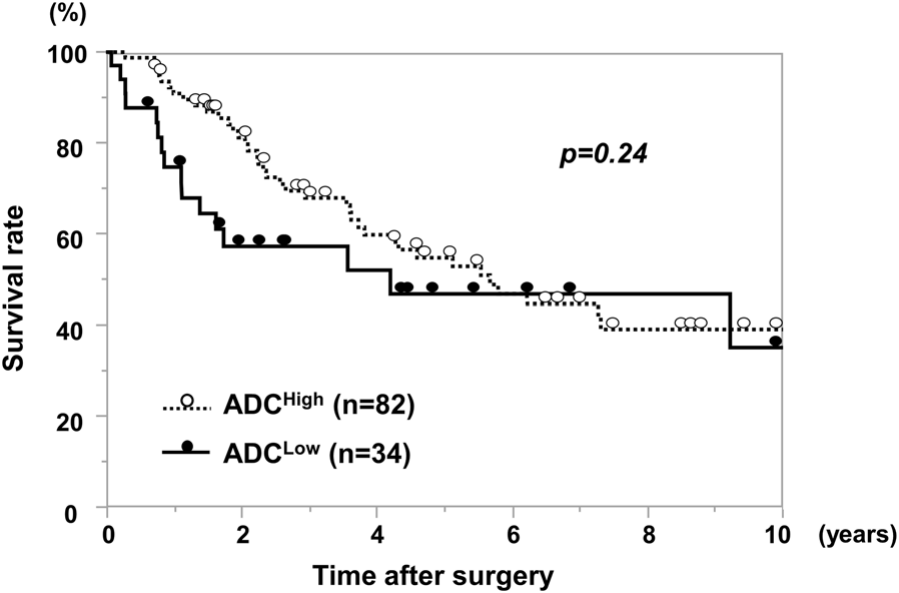
Disease-free survival rate of ADC^Low^ and ADC^High^ groups. There was no significant difference between the two groups

**Fig. 5:**
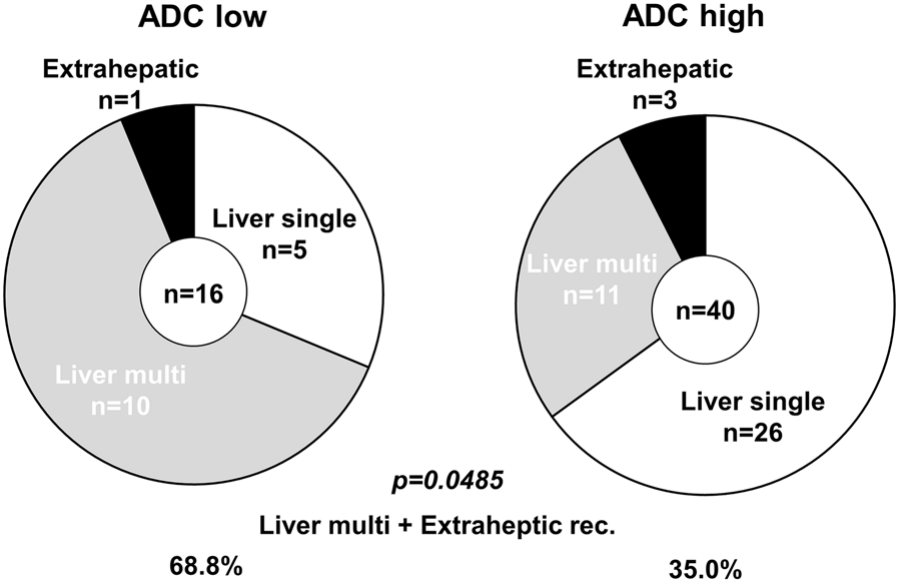
Recurrent patterns of ADC^Low^ and ADC^High^ groups. The ADC^Low^ group showed significantly higher rates of multiple liver and extra-hepatic recurrence (p < 0.05)

**Fig. 6:**
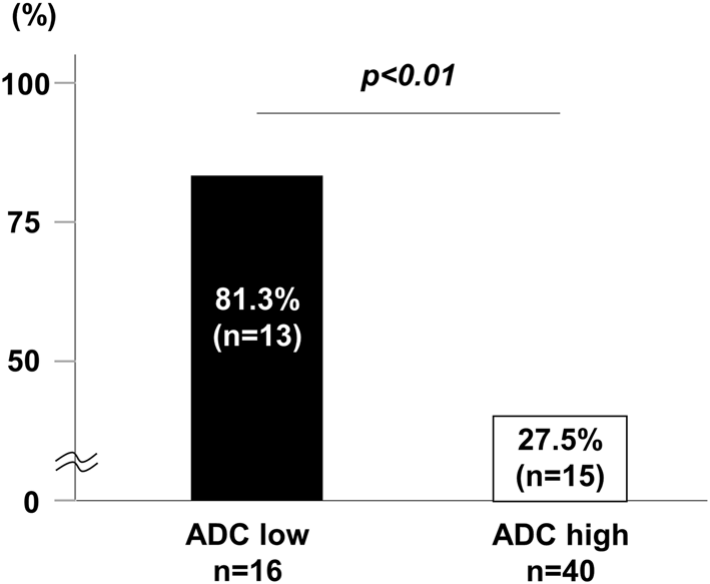
Rate of early recurrence within 2 years of surgery. The ADC^Low^ group showed higher rates of early recurrence compared with the ADC^High^ group (p < 0.01)

The OS rate of HCC patients after hepatectomy in the ADC^Low^ group was significantly worse than that in the ADC^High^ group (p < 0.01; Fig. 3). Three-year OS rates in the ADC^Low^ group and in the ADC^High^ group were 81% and 98%, respectively. Univariate analysis of OS identified high PIVKA-II (≥ 400 mAU/ml), multiple tumors, infiltrative growth pattern, and low ADC values as prognostic OS factors (p < 0.1). In multivariate analysis, infiltrative growth pattern and low ADC values were identified as independent prognostic factors (p < 0.05; Table 2). DFS rates after hepatectomy (Fig. 4) did not significantly differ between the two groups (p = 0.24). However, regarding recurrence patterns, the rate of multiple liver and extrahepatic recurrence was significantly higher in the ADC^Low^ group compared with the ADC^High^ group (68.8% versus 35.0%, p < 0.05; Fig. 5). Furthermore, early recurrence rates within 2 years after surgery were significantly higher in the ADC^Low^ group compared with the ADC^High^ group (81.3% versus 27.5%, p < 0.01; Fig. 6).

**Table 2:** Multivariate analysis for overall survival

Variable	Three-year survival rate (%)	Univariate	Multivariate	
		*p*-value	HR (95% C.I.)	*p*-value
Age (< 70/≥70 years)	95.8/91.6	0.83		
Sex (M/F)	95.9/88.2	0.80		
AFP (< 100/≥100 ng/ml)	94.1/92.3	0.29		
PIVKA-II (< 400/≥400 mAU/ml)	96.2/83.7	0.04	1.94 (0.69–5.43)	0.21
Tumor diameter (< 3/≥ 3 cm)	92.4/94.8	0.24		
Tumor number (single/multiple)	96.5/85.9	0.09	1.91 (0.67–5.45)	0.22
Growth type (Eg/Ig)	96.1/80.9	< 0.01	3.64 (1.42–9.30)	< 0.01
Portal invasion (−/+)	93.6/100	0.52		
ADC (high/low)	98.4/81.8	< 0.01	3.49 (1.39–8.79)	< 0.01

## Discussion

In this study, we demonstrate the potential role of ADC values in prognostic prediction for patients with early HCC after surgery: BCLC stage 0 and A. Despite therapeutic improvement, the recurrence rate after treatment for HCC remains high [18]. About half of the patients in this study developed tumor recurrence. Preoperative methods that predict prognosis in patients with early HCC are essential for establishing an appropriate therapeutic strategy, and help determine more aggressive treatments for high-risk patients to decrease post-operative recurrence.

In this study, low ADC values correlated with higher PIVKA-II levels and higher rates of infiltrative growth pattern. PIVKA-II is now established as a powerful prognostic factor for HCC [20, 21]. Infiltrative growth pattern was found to be associated with worse prognosis related to hypoxic/fibrotic tumor microenvironment or high expression of stemness-related markers [22].

Regarding the mechanism of variation of ADC values, progression of HCC is associated with increased cellular atypia in viable tumor, such as abnormal mitotic activity and nucleus/cytoplasm ratio [23]. These factors could theoretically reduce free diffusion of water molecules within the intracellular space, and lead to reduced ADC values [24]. However, microscopic or macroscopic tumor necrosis results in increased ADC values [25]. These tumor characteristics could influence ADC values associated with HCC. Lee et al. [15] report that low ADC values were associated with early recurrence of HCC after surgery. Mori et al. [16] showed that low ADC values correlate with poorly differentiated tumor, micro vessel invasion, and metastatic recurrence. However, no studies have reported prognostic prediction of overall survival using ADC for early HCC of BCLC stage 0 and A after surgery. Our study also demonstrated that low ADC values are associated with progressive tumor status. Although DFS showed no significant difference between ADC^High^ and ADC^Low^ groups, patterns of early and aggressive recurrence characterized the ADC^Low^ group, which might indicate poor prognosis in overall survival.

Preoperative non-invasive prediction of aggressive HCC characteristics based on imaging technology is very important for decreasing recurrence after surgical resection. Low ADC values may help surgeons select appropriate surgical procedures, such as wide resection margin, or liver transplantation. Furthermore, HCC patients with low ADC values should be followed up intensively after surgery [15]. Regarding other methods for prognostic prediction, Li et al. [26] reported that fluorodeoxyglucose (FDG) positron emission tomography (PET) and computed tomography (CT) could estimate microvascular invasion and DFS. However, PET/CT is not common for preoperative assessment of HCC. MRI is more commonly used for preoperative assessment, and has the advantage of combining tumor detection, prediction of liver function, and preoperative simulation [27].

The present study has several limitations. First, selection bias might exist because we retrospectively analyzed only patients with HCC who underwent MRI. Second, patients in our study were enrolled in only one center, and the number of cases was relatively small. Thus, further research with a larger, prospectively collected population is warranted to confirm these results.

## Conclusion

ADC values from DWI-MRI might be useful for prognostic prediction of patients with early HCC: BCLC stage 0 and A.

## Data Availability

The datasets generated and/or analyzed during the current study are available from the corresponding author on reasonable request.

## Abbreviations

BCLC: Barcelona clinic liver cancer
ADC: Apparent diffusion coefficients
DWI: Diffusion-weighted images
MRI: Magnetic resonance imaging
HCC: Hepatocellular carcinoma
PIVKA-II: Protein induced by vitamin K absence-II
OS: Overall survival
DFS: Disease-free survival
AFP: Alpha-fetoprotein
PIVKA-II: Protein induced by vitamin-K absence II
Eg: Expansive growth
Ig: Infiltrative growth
HR: Hazard ratio
